# TLR agonists as vaccine adjuvants in the prevention of viral infections: an overview

**DOI:** 10.3389/fmicb.2023.1249718

**Published:** 2023-12-21

**Authors:** Mohammad Enamul Hoque Kayesh, Michinori Kohara, Kyoko Tsukiyama-Kohara

**Affiliations:** ^1^Department of Microbiology and Public Health, Faculty of Animal Science and Veterinary Medicine, Patuakhali Science and Technology University, Barishal, Bangladesh; ^2^Department of Microbiology and Cell Biology, Tokyo Metropolitan Institute of Medical Science, Tokyo, Japan; ^3^Transboundary Animal Diseases Centre, Joint Faculty of Veterinary Medicine, Kagoshima University, Kagoshima, Japan

**Keywords:** TLR, hepatitis virus, HIV, SARS-CoV-2, influenza virus

## Abstract

Tol-like receptor (TLR) agonists, as potent adjuvants, have gained attention in vaccine research for their ability to enhance immune responses. This study focuses on their application in improving vaccine efficacy against key viral infections, including hepatitis B virus (HBV), hepatitis C virus (HCV), human immunodeficiency virus (HIV), SARS-CoV-2, influenza virus, and flaviviruses, including West Nile virus, dengue virus, and chikungunya virus. Vaccines are crucial in preventing microbial infections, including viruses, and adjuvants play a vital role in modulating immune responses. However, there are still many diseases for which effective vaccines are lacking or have limited immune response, posing significant threats to human health. The use of TLR agonists as adjuvants in viral vaccine formulations holds promise in improving vaccine effectiveness. By tailoring adjuvants to specific pathogens, such as HBV, HCV, HIV, SARS-CoV-2, influenza virus, and flavivirus, protective immunity against chronic and emerging infectious disease can be elicited.

## Introduction

Viral infections pose a significant threat to human health, and while vaccines exist for many viruses, there are still numerous viruses without approved vaccines. Additionally, the limited treatment options for viral infections, as antibiotics are ineffective against viruses, further highlight the importance of viral vaccines in prevention. Developing safe and efficient vaccines to combat viral infections is of utmost importance. Adjuvants, substances used in vaccines to stimulate and enhance immune response, have gained significant research interest for improving vaccine effectiveness and durability (Pulendran and O'hagan, 2021).

The innate immune system is a key component of host immunity that plays a significant role in the host defense against invading pathogens, including viruses (Medzhitov and Janeway, 2000). Germ-line encoded pattern recognition receptors (PRRs), including Toll-like receptors (TLRs), nucleotide oligomerization domain (NOD)-like receptors (NLRs), and retinoic acid-inducible gene-I (RIG-I)-like receptors (RLRs) recognize conserved structures of microbes, which are known as pathogen-associated molecular patterns (PAMPs), directly activate immune cells (Kawai and Akira, 2009; Mogensen, 2009; Wicherska-Pawlowska et al., 2021). TLRs are transmembrane receptors that can be found both on the cell surface and in intracellular membranes, and NLRs and RLRs are intracellular receptor molecules (Wicherska-Pawlowska et al., 2021). Along with TLRs, NLRs and RLRs also play significant role in the recognition of different viruses, including hepatitis B virus (HBV), hepatitis C virus (HCV), influenza virus, human immunodeficiency virus (HIV), Zika virus, measles virus, etc. and subsequently cause innate immune activation for shaping adaptive immunity (Kato et al., 2006; Wicherska-Pawlowska et al., 2021).

**Figure 1:**
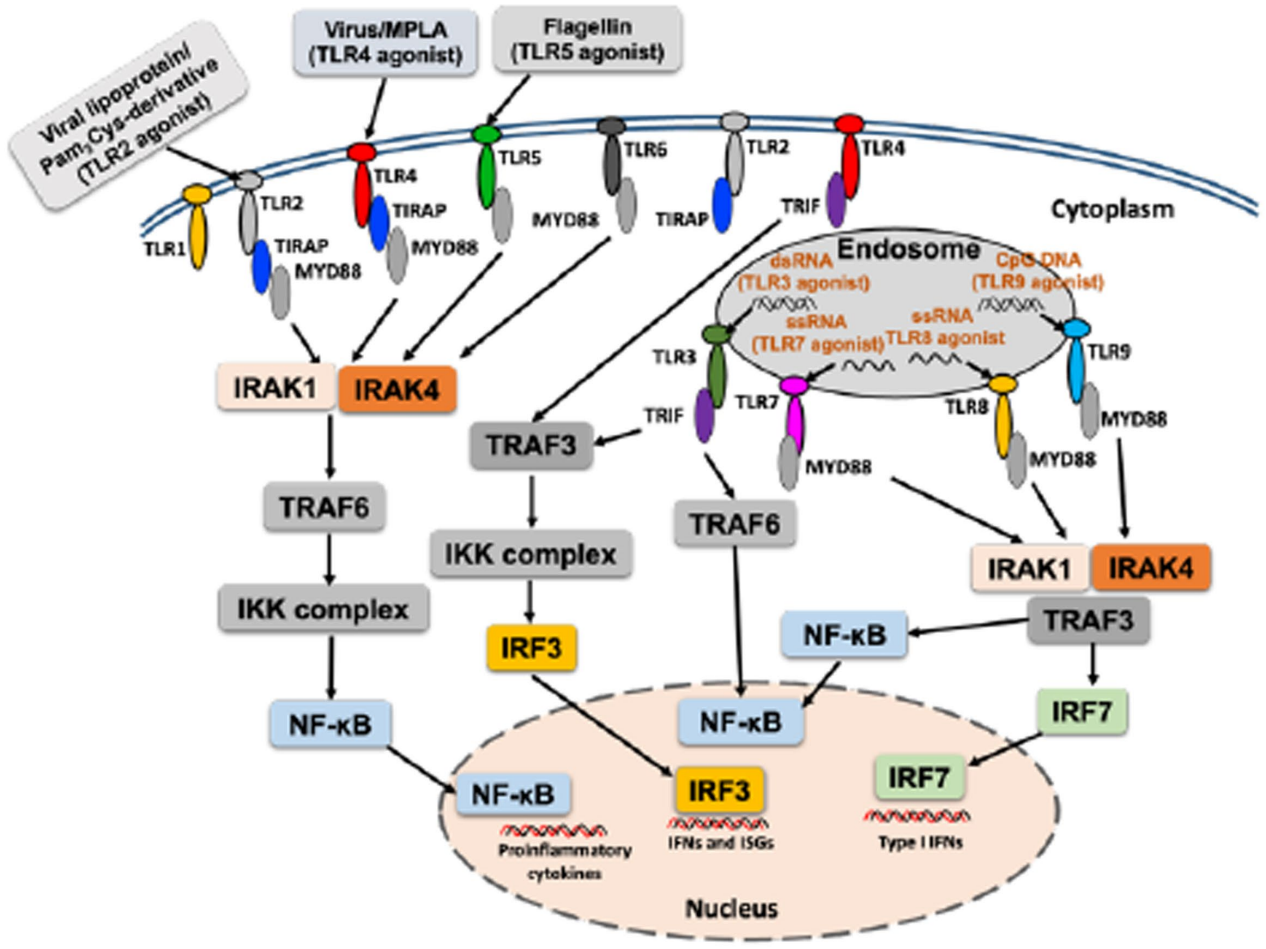
An overall mechanism of activation of TLR with respective TLR agonist.

Toll-like receptors (TLRs) are the best studied PRRs, playing a key role in sensing PAMPs and induce immune responses that shape adaptive immunity (Janeway and Medzhitov, 2002; Kayesh et al., 2021c). To date, 10 TLRs (TLR1–TLR10) have been identified in humans, and 13 in mice, and different studies have revealed their respective TLR ligands (Takeda and Akira, 2005). Respective TLR agonist can activate specific TLR signaling, and following recognition of ligands, TLRs recruit adapter molecules such as myeloid differentiation primary response protein 88 (MyD88), TIR domain-containing adapter protein (TIRAP)/MyD88 adaptor–like protein (MAL), Toll–IL-1–resistance (TIR) domain-containing adapter inducing IFN-β (TRIF; also known as TICAM1) and TRIF-related adapter molecule (TRAM; also known as TICAM2) culminate in the activation of nuclear factor (NF)-κB or interferon (IFN) regulatory factor (IRF), regulating immunomodulation (Takeda and Akira, 2005; Kawai and Akira, 2006; Figure 1). MyD88 is the common downstream adaptor recruited by all TLRs, except for TLR3 (Takeuchi et al., 2000; Figure 1). TLRs activate multiple steps in the induction of inflammatory reactions toward eliminating the invading pathogens and help in the systemic defense (Iwasaki and Medzhitov, 2004). A strong activation of the innate immune system is important for maturation and activation of immune cells as well as production of cytokines and chemokines to induce a potent adaptive immune response (Edwards et al., 2017). Moreover, TLRs play role in multiple dendritic cell functions and induce signals that are critical for initiation of the adaptive immune responses (Iwasaki and Medzhitov, 2004). For further details on TLR signaling that culminate in the production of cytokines/chemokines, please see other published reviews (Kawai and Akira, 2006; Brown et al., 2011; Duan et al., 2022).

Numerous investigations are currently underway to develop an effective adjuvant system using TLR agonists to enhance vaccine efficacy (Dowling, 2018; Kayesh et al., 2021a,c; Pulendran and O'hagan, 2021). TLRs are a family of type I transmembrane receptors containing three domains: an N-terminal extracellular leucine-rich repeat domain that recognizes specific PAMPs, a single transmembrane domain, and an intracellular Toll-interleukin 1 receptor (TIR) domain required for downstream signal transduction (Kawai and Akira, 2010).

Agonists come from a variety of sources, both natural and synthetic, and specific natural ligands have been identified for different TLRs. Examples include lipoproteins and peptidoglycans for TLR2, double-stranded RNA of viral origin for TLR3, bacterial lipopolysaccharide (LPS) and lipoteichoic acid for TLR4, bacterial flagellin for TLR5, single-stranded RNA for TLR7 and TLR8, unmethylated CpG motifs found in bacterial DNA or viruses for TLR9, and viral protein/viral RNP complexes for TLR10 (Weeratna et al., 2005; Lee et al., 2014; Bezemer and Garssen, 2020; Fore et al., 2020). Synthetic TLR agonists, developed by mimicking the molecular patterns and the immunostimulatory activities of their natural ligands, have been investigated for specific applications (Yang et al., 2022). Synthetic TLR agonists, such as Pam_3_CSK_3_ for TLR2 and TLR6, Poly I:C for TLR3, monophosphoryl lipid A (MPLA) for TLR4, imidazoquinoline-like molecules, imiquimod (R-837), resiquimod (R-848), S-27609, and guanosine analogs (e.g., loxoribine) for TLR7/8, as well as unmethylated CpG DNA for TLR9, have been reported (Gorden et al., 2005; Kaczanowska et al., 2013).

Cyclic dinucleotides (CDNs), including cyclic di-adenosine monophosphate, cyclic di-guanosine monophosphate, cyclic GMP-AMP (cGAMP) are ubiquitous small molecule second messengers synthesized by bacteria, which can activate different immune pathways, including the stimulator of interferon genes (STING; Dubensky et al., 2013; Gogoi et al., 2020). CDNs have been used as a relatively new class of vaccine adjuvants in many vaccine formulations, and shown to increase potency of bacterial vaccines (Ogunniyi et al., 2008; Hu et al., 2009; Yan et al., 2009). In addition, it has been shown that CDNs-adjuvanted subunit vaccine can elicit both Th1 and Th17 immune response, providing long-lasting protective immunity to *Mycobacterium tuberculosis* in mouse model (Van Dis et al., 2018). Therefore, the investigation of CDNs as an adjuvant in the viral vaccines could also be promising.

For an effective immunization approach, an effective vaccine is required, capable of generating long-lasting high titers of neutralizing antibodies with minimal antigen content. An important factor in meeting these requirements is the selection of an appropriate adjuvant. Until recently, alum was the only adjuvant approved for human use in the United States (Baylor et al., 2002). However, in 2009, the U.S. Food and Drug Administration granted licensure for human use to the first TLR ligand-vaccine, a TLR4 agonist-adjuvanted human papillomavirus vaccine (Centers for Disease Control and Prevention, 2010). Accordingly, numerous studies are focused on developing vaccines with novel adjuvant formulations for refining or developing vaccines against various infections (Reed et al., 2013).

Notably, the selection of an appropriate vaccine adjuvant is critical for effective vaccine efficacy, which plays an important role in augmenting the immune response against particular pathogens (Reed et al., 2013). Although actual mechanisms of adjuvants remain poorly understood, several potential mechanisms have been proposed, including the formation of depots for slow antigen release, activation of immune cells and antigen processing, and induction of cytokines and chemokines, all of which contribute to enhancing vaccine effectiveness (Awate et al., 2013). Adjuvants can influence vaccine response through various means, such as increasing functional antibody titers, increasing effector T cells, facilitating the rapid induction of protective responses, reducing antigen dosage and number of injections, enhancing memory and persistence (B and T cells), boosting responses in the immunocompromised individual, and broadening the scope of the immune response (Maisonneuve et al., 2014).

In modern vaccines, subunit components of pathogens are frequently used for vaccine preparation instead of employing whole, killed, or attenuated pathogens. However, this approach may reduce immunogenicity, requiring vaccine adjuvants to be incorporated with the antigens to enhance the immune response (Luchner et al., 2021). Aluminum-containing adjuvants, the first human vaccine adjuvants approved in clinical use, induce strong humoral immunity but do not equally stimulate cellular immunity, rendering them ineffective against intracellular virus infections (Igietseme et al., 2004; Oleszycka et al., 2018; Wang and Xu, 2020). There is an increasing interest in the use of TLR agonists as immunomodulators that can influence the outcome of treatment of infection (Kayesh et al., 2021a,c; Jimenez-Leon et al., 2023). TLR agonists have shown high potency in activating innate immunity in a number of vaccine adjuvants and immunomodulatory agents against infectious diseases and cancers (Luchner et al., 2021). The use of TLR agonists as vaccine adjuvants remains the current focus of research aimed at increasing vaccine efficacy. In this study, we review the progress and useability of TLR agonists as vaccine adjuvants in different viral vaccines, particular focusing on hepatitis B virus (HBV) and SARS-CoV-2 vaccines, and other vaccines, including hepatitis C virus (HCV), human immunodeficiency virus (HIV), influenza virus, and flavivirus [dengue virus (DENV), West Nile virus (WNV), Chikungunya virus (CHIKV)] vaccines.

## Prospects of TLR agonists in viral vaccines

TLR agonists are extensively studied as adjuvants to enhance vaccines effectiveness (Pulendran and O'hagan, 2021). These agonists have gained significant attention as potent immunomodulators capable of inducing the production of IFN, proinflammatory cytokines, and chemokines. They show promise against various viral infections, including HBV, HCV, HIV-1, influenza virus, and SARS-CoV-2 (Martinsen et al., 2020; Kayesh et al., 2021a,c, 2022, 2023; Jimenez-Leon et al., 2023; Yin et al., 2023). However, further studies are needed to identify potential TLR agonists for vaccine adjuvants. For example, a recent study compared three TLR agonists [TLR1/2 (Pam3Cys), TLR7/8 (R848), or TLR9 (CpG ODN)] as vaccine adjuvants in combination with inactivated porcine reproductive and respiratory syndrome virus (iPRRSV). The study found no detectable antigen-specific immune response after intramuscular (i.m.) or subcutaneous vaccination. However, a non-antigen-specific IFN-γ production was observed by the TLR9 agonist group, which reduced viremia upon challenge compared to that in the non-vaccinated animals (Vreman et al., 2019).

The development of effective peptide and DNA vaccines is hindered by poor immunogenicity. To address this challenge, researchers are exploring novel adjuvants, including TLR agonists, which offer immunomodulating and immunotherapeutic effects (Surendran et al., 2018; Mullins et al., 2019). TLR stimulation mediates T_H_1 and T_H_17 immune responses (Agrawal et al., 2003), and the synergistic activation of TLRs and NLRs has been reported to elicit these immune responses (Fritz et al., 2007; Magalhaes et al., 2008, 2011). The increasing understanding of TLR agonists has captured the attention of vaccinologists (Dowling, 2018), and the COVID-19 pandemic further accelerated clinical research on vaccines incorporating TLR agonist-based adjuvants (Yang et al., 2022). The inclusion of TLR agonists in vaccine development holds promise for enhancing vaccine efficacy.

## TLR agonists as vaccine adjuvants in HBV vaccines

Despite the availability of an effective preventive vaccine for HBV, chronic HBV infection remains a global health problem (Ezzikouri et al., 2020). According to the World Health Organization (WHO), an estimated 296 million people were affected by chronic HBV infection in 2019, with 1.5 million new infections occurring annually (World Health Organization, 2022). While the currently available recombinant HBV vaccines demonstrate excellent safety and immunogenicity (Assad and Francis, 1999), they still face challenges in inducing protective immunity in poor vaccine responders, including immunocompromised people, older adults, diabetics, and those with chronic kidney disease (Lee and Lim, 2021).

Immune tolerance poses a significant obstacle in the cure of HBV infection (Tran, 2011). The mechanism behind immune tolerance is not well understood, but HBV-specific T-cell hyporesponsiveness may impair antigen processing and transport to major histocompatibility complex class I molecules (Sukriti et al., 2010). In an investigation of overcoming HBsAg-specific immune tolerance state in humanized mice model, immunization with CL097 (TLR7/8 agonist)-conjugated HBV-Ag was found to reverse immune tolerance in HBV-Tg mice, and induced antigen-specific immune responses (Wang et al., 2014). TLR7/8 agonists demonstrated potent adjuvant properties in inducing antigen-specific Th1 responses in an immune tolerant state (Wang et al., 2014). In the woodchuck model of chronic HBV, GS-9688, an oral selective small molecule agonist of TLR8, effectively reduced serum viral load by over five logs and also suppressed woodchuck hepatitis surface antigen in 50% of treated woodchucks (Daffis et al., 2021). Another study showed that GS-9688 induced cytokines in human peripheral blood mononuclear cells, leading to the activation of antiviral effector function by increasing the frequency of HBV-specific CD8+ T cells, CD4+ follicular helper T cells, NK cells, and mucosal-associated invariant T cells (Amin et al., 2021). In our previous study, we observed that HBV infection in tree shrew model could induce a significant suppression of IFN-β response at 31 weeks post-infection, contributing to the chronicity (Kayesh et al., 2017a). In addition, no induction of TLR3 and suppression of TLR9 was found (Kayesh et al., 2017a).

HEPLISAV-B is a recombinant HBV vaccine composed of HBsAg combined with the CpG 1,018 adjuvant, which stimulates innate immunity through TLR9 (Lee and Lim, 2021). In a Phase III clinical trial, HEPLISAV-B demonstrated the ability to rapidly and consistently produce high titers of sustained seroprotection with fewer immunizations, including in individuals with poor response to vaccination (Eng et al., 2013). Compared to Engerix-B, HEPLISAV-B has shown superior and earlier seroprotection, while maintaining a favorable profile (Lee and Lim, 2021). Notably, HEPLISAV-B was found to induce higher seroprotection rates in the poor vaccine responders such older adults, diabetics, and those with chronic kidney disease, surpassing the effectiveness of Engerix-B (Lee and Lim, 2021). This suggests that HEPLISAV-B is more efficacious in these individuals. Historically, persons living with HIV (PLWH) have exhibited poor responses to HBV vaccination, with low seroprotection rates ranging from 35% to 70% following a 3-dose series. However, according to a press release regarding the study findings of an ongoing phase 3 clinical trial (ClinicalTrials.gov, ID: NCT04193189) presented by the National Institute of Allergy and Infectious Diseases (NIAID), HEPLISAV-B, an HBV vaccine containing recombinant HB surface antigen and a CpG-based TLR9 agonist, has been found to be effective in protecting adults living with HIV who have never been vaccinated against or infected with HBV (National Institutes of Health, 2022).

Adjuvant System 04 (AS04) combines aluminum hydroxide with the TLR4 agonist 3-O-desacyl-4′-monophosphoryl lipid A (Didierlaurent et al., 2009). FENDrix (GlaxoSmithKline Biologicals), an adjuvanted HBV vaccine consists of recombinant HBsAg formulated with aluminum phosphate and MPL, a purified, detoxified derivative of the lipopolysaccharide molecule found in the bacterial wall of *Salmonella minnesota* (Garcon et al., 2007). This vaccine has been investigated for its ability to elicit a better immune response. FENDrix is administered in a four-dose scheme: on day 0, month 1, month 2, and month 6 (after day 0). Due to the improved adjuvant system, FENDrix induces higher concentrations of protective antibodies more rapidly (Kundi, 2007). In a prospective cohort study, HB-AS04 showed a higher efficacy in patients on maintenance dialysis, although a significant number of non-responders were still present (Fabrizi et al., 2015). Another open-label, non-randomized trial showed the persistence of anti-HBs antibody among responder patients during a long follow-up period (Fabrizi et al., 2020). In a multicenter phase 3 comparative study involving adults receiving hemodialysis who had previously received HBV vaccination but were not seroprotected, a booster dose of HEPLISAV-B was found to induce a higher seroprotection rate (52.8%; 95% confidence interval [CI]: 38.6, 66.7%) compared to Engerix-B (32.6%; 95% CI: 19.5, 48.0%) and FENDrix (43.1%; 95% CI: 29.3, 57.8%) recipients (Girndt et al., 2022).

HIV/HBV co-infection may lead to increased morbidity and mortality compared to HBV or HIV mono-infection (Whitaker et al., 2012; Kayesh et al., 2023). Although the immunogenicity of HBV vaccines is impaired in HIV-infected patients (de Vries-Sluijs et al., 2020), immunization with a HBV vaccine is the most effective way to prevent infection in people with HIV. To enhance the vaccine response in immunocompromised individuals, new strategies are needed, such as the addition of new adjuvants or increased vaccine doses (Whitaker et al., 2012; Catherine and Piroth, 2017). A randomized controlled trial in HIV-infected patients revealed an insignificant (*p* = 0.09) increase in anti-HBs ≥ 10 IU/L response rate at week 28 following FENDrix (85.7%) and Engerix (65.0%) vaccination (de Vries-Sluijs et al., 2020). Notably, a recent multicenter open-label study of TLR9 agonist-adjuvanted HEPLISAV-B vaccine in HIV-positive individuals without prior HBV vaccination found that all 68 participants achieved HBV seroprotective titers after the 3-dose series in the primary analysis, with no unexpected safety concerns (Marks et al., 2023). These findings highlight the increased immunogenicity of HBV vaccines and the immunomodulator potential of TLR agonists in enhancing vaccination efficacy. The TLR agonist adjuvants currently under development for HBV vaccines are listed in Table 1.

**Table 1:** TLR agonists as vaccine adjuvants in HBV vaccines.

Vaccine name	Sponsor/company	TLR agonist adjuvant	Target TLR	Clinical phase	Disease target	Effects on host immunity	Clinical Trials. Gov identifier/reference
HEPLISAV-B	Dynavax Technologies Corporation	CpG 1,018	TLR9	Phase III	Hepatitis B	Strongly favors development of the Th1 subset of helper T cells	Hyer and Janssen (2018)
HEPLISAV-B	Dynavax Technologies Corporation	CpG 1,018	TLR9	Phase III	Hepatitis B (HIV coinfection)	Induces seroprotective titers in all participants with HIV without prior HBV vaccination	Marks et al. (2023)
HBV-AS04	GlaxoSmithKline	Monophosphoryl-Lipid A (MPLA)	TLR4	Phase III	Hepatitis B	Improves seroresponse	Fabrizi et al. (2015, 2020)

## TLR agonists as vaccine adjuvants in HCV vaccines

HCV causes chronic liver infection and is a leading cause of liver cancer. According to the WHO, 58 million people worldwide are chronically infected with HCV, with an annual approximately 1.5 million new infections [World Health Organization (WHO), 2022]. Although newly approved direct-acting antivirals (DAAs) have shown great therapeutic success for HCV infection (Li and De Clercq, 2017), DAA therapy is costly and often results in side effects, limiting its accessibility to patients. Notably, DAA therapy has been associated with an increasing risk of hepatocellular carcinoma (HCC) in patients treated with DAAs (Chinchilla-Lopez et al., 2017; Tajiri et al., 2022). Currently, there is no licensed protective vaccine against HCV, making the development of an effective preventive vaccine is critical (Behmard et al., 2022).

Scientists are diligently working on different strategies to develop an effective HCV vaccine. Immunoinformatics-based multi-epitope constructs, along with the use of TLR3 and TLR4 agonists, have shown immunogenicity, non-allergenicity, and non-toxicity (Behmard et al., 2022), requiring further investigation into the protective traits and safety of these designed candidates. The improved efficacy of HCV vaccine candidates due to TLR agonists has been reported in several studies. In a phase 1 study, TLR9 agonist CpG 10,101 was found to dose-dependently increase immune marker activation while simultaneously decreasing HCV RNA levels (McHutchison et al., 2007), supporting the future exploration of CpG 10,101 as a vaccine adjuvant for HCV candidates. Furthermore, including TLR7 and TLR9 agonists in HCV vaccine candidates has been shown to promote the maturation of plasmacytoid dendritic cells, leading to improved antigen presentation and enhanced viral immunization (Dominguez-Molina et al., 2018). In an earlier study, an induction of TLR3, TLR7 and TLR8 mRNA was observed in HCV-infected tree shrew liver, compared to uninfected liver tissues (Kayesh et al., 2017b).

HCV virus-like particle (VLP)-based vaccines adjuvanted with TLR2 agonist Pam(2)Cys [E(8)Pam(2)Cys] induced significant HCV-LP and E2-specific antibody responses mice. In comparison to traditionally alum-adjuvanted VLPs, a single dose of VLPs formulated with this lipopeptide achieved antibody titers equivalent to those obtained with up to three doses of traditionally alum-adjuvanted VLPs (Chua et al., 2012).

## TLR agonists as vaccine adjuvants in HIV vaccines

Developing a HIV-1 vaccine that can generate high titers of functional antibodies against HIV-1 remains a high priority. Single or combined effect of TLR agonists is now also being investigated for the development of TLR agonist-adjuvanted HIV-1 vaccines (Moody et al., 2014; Rozman et al., 2023). It has been shown that HIV Gag protein conjugated to TLR7/8 agonist (3 M-012) could enhance the magnitude of Th1 and CD8+ T cell responses in nonhuman primates (Wille-Reece et al., 2005). In a rhesus macaque model, it has been shown that TLR agonists can enhance epitope-specific HIV-1 Env reactive antibody levels (Moody et al., 2014). It was also observed that the combination of TLR7/8 and TLR9 agonists could elicit higher titers of neutralizing and ADCC-mediating antibodies (Moody et al., 2014).

Studies have shown that combining ligands for three TLRs (TLR2/6, TLR3, and TLR9) can increase the production of DC IL-15, promoting DC activation and stimulation of NK cells (Mattei et al., 2001; Anguille et al., 2015). This combination has also greatly increased the protective efficacy of HIV envelope peptide vaccines in mice models (Zhu et al., 2010).

A recent study reported that intranasal administration of TLR7/NOD2L agonist in conjunction with the NP-p24 HIV vaccine resulted in a potent adjuvant effect, inducing high-quality humoral and adaptive immune responses both in systemic and mucosal compartments (Gutjahr et al., 2020). Long-lived plasma cells (LLPCs), primarily residing in the bone marrow, are critical mediators of durable antibody responses (Liu et al., 2022). It has been reported that 3 M-052, a TLR7- and TLR8-agonist adjuvant can induce notably high and persistent (up to ~1 year) frequencies of Env-specific LLPCs in the bone marrow and serum antibody responses in rhesus macaques (Kasturi et al., 2020). A recent study showed that TLR4 agonist-based nanoparticle adjuvant, saponin/MPLA nanoparticles (SMNP) can enhance lymph flow and antigen entry into lymph nodes in animal models (Silva et al., 2021). Silva et al. reported that a single dose vaccination with Env trimers combined with SMNP adjuvant could lead to seroconversion in all vaccinated male and female Indian rhesus monkeys (*Macaca mulatta*) with excellent HIV neutralizing antibody titers (Silva et al., 2021), whereas previous studies observed little or no Env-specific IgG in non-human primates after single immunizations of HIV Env trimers with various adjuvants, suggesting SMNP as a promising vaccine adjuvant candidate to be used for further studies for clinical use in HIV infection. TLR7, an endosomal receptor and nucleotide-binding oligomerization domain 2 (NOD2), a cytosolic receptor, are widely expressed at mucosal levels, functioning as key innate receptors (Gutjahr et al., 2020). It has been reported that chimeric TLR7/NOD2 agonist was highly potent to stimulate DC maturation both *in vitro* and *in vivo*. Intranasal administration of TLR7/NOD2L agonist with NP-p24 HIV vaccine was found effective in inducing both humoral and adaptive immune response in systemic and mucosal compartments (Gutjahr et al., 2020).

## TLR agonists as vaccine adjuvants in SARS-CoV-2 vaccine

Respiratory virus infections, such as SARS-CoV-2, remain a major global human health concern requiring appropriate preventive measures. Despite success in vaccinating populations against SARS-CoV-2 infection, there are still concerns that need to be addressed, such as duration of immunity, efficacy against emerging variants, protection from infection and transmission, and worldwide availability of vaccines (Atalis et al., 2022). To tackle these issues related to SARS-CoV-2 infection, the use of TLR agonists as vaccine adjuvants can be investigated. Studies have shown that intranasal administration of the TLR2/6 agonist INNA-051 in ferret model significantly reduced SARS-CoV-2 viral RNA levels in the nose and throat (Proud et al., 2021). TLR agonists are potent immunomodulators that can improve and broaden the efficacy and durability of vaccine responses (Pulendran and O'hagan, 2021), making them valuable in the development of potent SARS-CoV-2 vaccines.

Furthermore, SARS-CoV-2 subunit vaccines adjuvanted with TLR4 and RIG-I agonists have demonstrated the ability to induce robust and unique route-specific adaptive immune responses against SARS-CoV-2 (Atalis et al., 2022). SARS-CoV-2 spike subunit vaccine adjuvanted with a dual TLR ligand liposome induced robust systemic neutralizing antibodies in a mouse model of COVID-19, and completely protected against a lethal SARS-CoV-2 challenge (Abhyankar et al., 2021). Another study reported varying degrees of protection based on different adjuvant platform against SARS-CoV-2 in rhesus macaques. AS37, a TLR7 agonist adsorbed to alum, and AS03, an α-tocopherol-containing oil-in-water emulsion, induced substantial neutralizing antibody titers in rhesus macaque, promoting protective immunity against SARS-CoV-2 (Arunachalam et al., 2021). These findings highlight the need for further research to identify an ideal adjuvant for SARS-CoV-2 vaccine.

A recent study reported that TLR7-nanoparticle (TLR7-NP)-adjuvanted influenza and SARS-CoV-2 subunit vaccines induced broad neutralizing antibodies in a mouse model, which protected against respective multiple viral variants (Yin et al., 2023). Importantly, the TLR7-NP adjuvant can induce cross-reactive antibodies targeting both dominant and subdominant epitopes and antigen-specific CD8+ T-cell responses in mice (Yin et al., 2023). Intranasal vaccination with CpG nanoparticle-adjuvanted HA influenza vaccine has been shown to increase protective efficacies in mice (Dong et al., 2022). Comparatively, PEI-HA/CpG nanoparticles generated more robust and balanced IgG1/IgG2a neutralizing antibody responses and Fc-mediated antibody-dependent cellular cytotoxicity, whereas PEI-HA nanoparticles primarily elicited IgG1-dominant antibody responses (Dong et al., 2022). Another study reported an overall magnitude of the immune response induced by SARS-CoV-2 spike glycoprotein (CoVLP) vaccine candidate adjuvanted with CpG 1,018 or AS03 (Ward et al., 2021).

Recent reports have highlighted the efficacy of TLR7 agonist-adjuvanted vaccines in inducing robust immune responses against SARS-CoV-2. Vaccination with the S1 subunit of the SARS-CoV-2 spike protein, adjuvanted with TLR7 agonist, has been shown to induce potent humoral and cellular immunity in mice. This approach resulted in a balanced Th1/Th2 immune response and effectively induced neutralizing antibodies against SARS-CoV-2 and all variants of concern (B.1.1.7/alpha, B.1.351/beta, P.1/gamma, B.1.617.2/delta, and B.1.1.529/omicron), suggesting a great potential of this adjuvant-protein conjugate vaccine candidate (Zhang et al., 2022). Another study also reported that a subunit SARS-CoV-2 vaccine with clinically relevant adjuvants such as alum, AS03 (a squalene-based adjuvant supplemented with α-tocopherol), AS37 (a TLR7 ligand emulsified in alum), CpG1018 (a TLR9 ligand emulsified in alum), O/W 1849101 (a squalene-based adjuvant) induced durable protection in mice. However, TLR-agonist-based adjuvants CpG1018 and AS37 induced Th1-skewed CD4+ T cell responses. In contrast, alum, O/W, and AS03 induced a balanced Th1/Th2 response (Grigoryan et al., 2022). Further supporting the potential of TLR agonists as adjuvants, a multicenter, double-blind, randomized, placebo-controlled trial demonstrated that SCB-2019 (30 μg, adjuvanted with 1·50 mg CpG-1018 and 0·75 mg alum) provided notable protection against the entire severity spectrum of COVID-19 caused by circulating delta, gamma, and mu variants of SARS-CoV-2 (Bravo et al., 2022). These findings highlight the promising role of TLR agonists as potential adjuvants. The TLR agonists adjuvants currently under development for SARS-CoV-2 vaccines are listed in Table 2.

**Table 2:** TLR agonists as vaccine adjuvants in SARS-CoV-2 vaccines.

Vaccine name	Sponsor/company	TLR agonist adjuvant	Target TLR	Clinical phase	Disease target	Effects on host immunity	Clinical Trials. Gov identifier/reference
SCB-2019	-	CpG-1018	TLR9	Phase II/III	COVID-19	Provides notable protection against all circulating delta, gamma and mu variants of SARS-CoV-2	Bravo et al. (2022)
SCB-2019 Recombinant SARS-CoV-2 Trimeric S-protein Subunit Vaccine	Zhejiang Clover Biopharmaceuticals, Inc.	CpG 1,018/Alum-adjuvanted	TLR9	Phase II	COVID-19	Results yet to be published	NCT04954131
CoVac-1 (COVID-19 peptide vaccine)	The University Hospital Tübingen	TLR 1/2 agonist XS15	TLR 1/2	Phase I	COVID-19	CoVac-1-induces IFN-γ T cell responses; induces broad, potent and variant of concern-independent T cell responses	Heitmann et al. (2022)
SARS-CoV-2 subunit vaccine	Georgia Institute of Technology, USA	MPLA	TLR4	Phase I	COVID-19	In mice model, MPLA+PUUC NPs enhanced CD4+ CD44+ activated memory T cell responses against spike protein in the lungs while MPLA NPs increased anti-spike IgA in the bronchoalveolar fluid and IgG in the blood	Atalis et al. (2022)
TLR7-NP adjuvanted SARS-CoV-2 vaccine	Stanford University, USA	Toll-like receptor 7 agonist nanoparticle (TLR7-NP)	TLR7	Phase I	COVID-19	Induces cross-reactive Abs for both dominant and subdominant epitopes and antigen-specific CD8+ T-cell responses in mice	Yin et al. (2023)

## TLR agonists as vaccine adjuvants in influenza virus vaccine

The development of more effective vaccines is required to combat influenza virus infection, and remains as a major goal of modern medical research. The use of TLR agonists in enhancing the influenza virus vaccine is under development. A reduced influenza-associated secondary pneumococcal infections has been reported in mice with co-administration of an inhaled TLR2 agonist with an inactivated vaccine (Hussell and Goenka, 2016), also highlight the effectiveness of TLR agonist use in influenza vaccine. Lopez et al. reported an enhanced vaccine efficacy with enhanced antibody response in case of Encevac TC4 vaccine administration along with TLR9 agonist, CpG oligodeoxynucleotides (Lopez et al., 2006). Flagellin, the structural component of bacterial flagella, is known as the TLR5 agonist that can cause the induction of cytokines and chemokines (Gewirtz et al., 2001; Lu and Sun, 2012). TLR5 also shows the potential to activate the immune cells and can initiate innate and adaptive immune response (Hajam et al., 2017). The use of bacterial flagellin as TLR5 agonist in viral vaccine appears promising (Hajam et al., 2017). Notably, flagellin has been extensively investigated as a mucosal adjuvant in epitope-based influenza vaccines, and appeared promising (Ben-Yedidia and Arnon, 2007; Song et al., 2008; Adar et al., 2009; Liu et al., 2011; Taylor et al., 2011).

In murine model, Goff et al. reported that recombinant hemagglutinin (HA) from the A/Puerto Rico/8/1934 strain (rPR/8 HA) in combination with TLR4 (1Z105, a substituted pyrimido[5,4-b]indole specific for the TLR4-MD2 complex) and TLR7 ligands (1 V270, a phospholipid-conjugated agonist) can induce rapid and sustained humoral immunity that is protective against lethal challenge with a homologous virus (Goff et al., 2015). Another study also showed that a dual combination of TLR4 and TLR7 ligands in recombinant influenza virus HA vaccine can induce a broader immune response (Sato-Kaneko et al., 2020). A previous study reported an enhanced efficacy of split-virus vaccines (SVVs)-mediated protection against influenza in older adults when combined with TLR4 agonist glucopyranosyl lipid adjuvant–stable emulsion (GLA-SE; Behzad et al., 2012). In a phase 2 clinical trial, an enhanced efficacy of H5N1 plant-made virus-like particle vaccine was observed when co-administered with GLA-SE (Pillet et al., 2018). A sustained polyfunctional and cross-reactive HA-specific CD4+ T cell response was observed in all vaccinated groups (Pillet et al., 2018).

Among the imidazoquinoline compounds, 1-benzyl-2-butyl-1H-imidazo[4,5-c]quinolin-4-amine (BBIQ) is a potential TLR7 agonist, and it was shown that recombinant influenza HA protein vaccine administered with BBIQ significantly enhanced anti-influenza IgG1 and IgG2c response in mice (Kaushik et al., 2020), suggesting BBIQ as a promising influenza vaccine adjuvant for further study. In another study, a licensed quadrivalent inactivated influenza vaccine (QIV) administered with RIG-I (SDI-nanogel) and TLR7/8 agonist (Imidazoquinoline) enhanced antibody and T cell responses, correlating with the protection against lethal influenza virus infection (Jangra et al., 2022). Clemens et al. reported that stem region of the HA adjuvanted with R848 (TLR7/8 agonist) could impact multiple cell types such as influenza-specific T follicular helper cells as well as Tregs that have the potential to contribute to the HA-stem response (Clemens et al., 2022). Overall, the use of TLR agonists in influenza vaccine might help in the development of more effective influenza vaccine in the near future.

## TLR agonists as vaccine adjuvants in vaccines against flavivirus infections, including West Nile virus, dengue virus, and chikungunya virus

TLR agonist adjuvants represent a promising tool toward enhancing the protective capacity of flavivirus vaccines and broadening of antiviral antibody responses with reduced dose and dosage (Van Hoeven et al., 2018). Currently there is no approved human vaccine for WNV. In a recent study, it has been shown that WNV recombinant E-protein vaccine (WN-80E) adjuvanted with TLR4 agonist SLA or the saponin adjuvant, QS21 was capable of inducing long-lasting immune responses in preclinical models with sterilizing protection in WNV challenge, reducing viral titers following WNV challenge to below detection levels in Syrian hamsters (*Mesocricetus auratus*; Van Hoeven et al., 2018). Flagellin has also been investigated as a mucosal adjuvant to be used in WNV recombinant protein vaccine with the induction of protective immune response (McDonald et al., 2007).

A DENV vaccine that is equally effective against all four serotypes is urgently needed. Toward the development of a panserotype dengue vaccine, the suitability of using TLR agonist as an adjuvant could be helpful (Kayesh et al., 2021b). A recent study investigated the immunogenicity and protective capacity of recombinant DENV NS1 administered with CDNs. It was observed that NS1-CDN immunizations could induce serotype-specific and cross-reactive antibody and T-cell responses in mice model. Further, NS1-CDN vaccinations showed efficacy in homotypic and heterotypic protection from DENV2-induced morbidity and mortality (Espinosa et al., 2019). It has been shown that dengue subunit vaccine consisted of recombinant DENV2 envelope domain III combined with TLR agonists induced strong immunological signatures involving immune cell trafficking, IFNs, and proinflammatory and T-cell responses, however, unexpectedly only partial protection was obtained against viral challenge (Bidet et al., 2019).

Until recently, there was no clinically approved CHIKV vaccine for immunization, however, on 9th November 2023, the U.S. Food and Drug Administration approved the first chikungunya vaccine, Ixchiq/VLA1553 (developed by Valneva Austria GmbH) for individuals 18 years of age and older who are at increased risk of exposure to CHIKV (U.S. Food and Drug Administration, 2023). In a multicenter, randomized, placebo-controlled phase 3 clinical study, CHIKV vaccine VLA1553 was found generally safe and equally well tolerated in younger and older adults that induced seroprotective chikungunya virus neutralizing antibody levels in 263 (98·9%) of 266 participants (Schneider et al., 2023). A recent study reported an enhanced efficacy of inactivated CHIKV-MPLA combination, which could induce higher neutralizing antibodies compared to unadjuvanted CHIKV vaccine (Gosavi and Patil, 2022). Although further studies are warranted, however, TLR4 agonist appears as a promising adjuvant candidate to be used for enhancing the efficacy of CHIKV vaccine (Gosavi and Patil, 2022).

## Discussion

Appropriate selection of specific pattern recognition receptor ligands (adjuvants) is critical for formulating the next generation vaccines, which will aim to induce an efficient adaptive immune response with minimal adverse reactions. Both the existing vaccines and new vaccine development could benefit from the use of TLR agonists as vaccine adjuvants, especially for viral vaccines targeting particular pathogens (Kayesh et al., 2021a). However, it is important to emphasize the need for comparative studies before selecting a TLR agonist as an adjuvant, and there is a currently a lack of research comparing the nature of immune responses induced by different candidate adjuvants.

## Limitation and future perspectives

Although TLR agonists appear as potent immune activators for immunomodulation, however, TLR activation-induced signaling may act as a double-edged sword, which may enhance immune-mediated pathologies instead of protection (Salaun et al., 2007; Huang et al., 2008; Yokota et al., 2010). Therefore, a clear understanding of TLR interactions with particular virus is critical for judicious use of TLR agonist in the vaccine. More preclinical studies are essential to perform for investigating the challenges of TLR use both in vaccines as well as in therapy. In addition, as combined use of TLR agonists is assumed to enhance the immune response, therefore, more future studies are warranted for investigating the combinatorial use of multiple TLR agonist and their effects on vaccine use (Albin et al., 2019). Also, the use of TLR agonist as vaccine adjuvants can be extended for investigation in other viral vaccines.

## Conclusion

The use of TLR agonists as vaccine adjuvant has revolutionized the modern vaccine science due to its potential in improving vaccine effectiveness. In addition, TLR agonist has opened up a new research window for enhancing efficacy of the existing vaccines as well as for developing the new vaccines. Many TLR agonist candidates are under investigation, and by proper tailoring of TLR agonists in vaccine formulation, the vaccine effectiveness can be improved that should help in protecting chronic and emerging viral diseases.

## Author contributions

MEHK, MK, and KT-K: conceptualization and writing—review and editing. MEHK and KT-K: writing—original draft preparation. All authors contributed to the article and approved the submitted version.
